# Presenting the Inner Triangle Outer Diamond (ITOD) Framework of Educational Ecosystems and Its Applications to the Integration of Educational Technologies and Innovations

**DOI:** 10.7759/cureus.99949

**Published:** 2025-12-23

**Authors:** Joshua Owolabi

**Affiliations:** 1 Biomedical Sciences, Philadelphia College of Osteopathic Medicine, Moultrie, USA

**Keywords:** ai, ecosystem, edtech, framework, innovation, medical education, simulation in medical education, technology

## Abstract

Educators are leveraging technologies and innovations, including artificial intelligence (AI), to support learners and deliver quality medical education. Many of these initiatives have reportedly yielded positive impacts. There have also been publications and reports highlighting the success of educational technologies (EdTech) and innovations, as well as their potential to not only enhance the outcomes of educational programs but also define the future of medical education and practice. Proponents of EdTech and innovations argue that the significant introduction of EdTech and innovations into programs can enhance learners' experiences, improve programme outcomes, and prepare learners for a future of work and practice expected to be significantly shaped by technologies and innovation. However, it is equally important to address the unintended consequences of poor technology application, particularly when approaches to introducing and deploying EdTech and innovations are not properly grounded in learning theories and pedagogical principles or are improperly integrated into the educational ecosystem. A major issue associated with improper deployment and poor integration of educational technologies and innovations is heterogeneity, in addition to other reported contextual challenges and limitations. This underscores the need to approach the introduction, deployment, and integration of EdTech and innovation from an ecosystem perspective, providing medical educators and other stakeholders with a comprehensive view of what constitutes the ecosystem supporting medical education. Such an approach ensures the optimization of technologies and innovations through systems thinking. The inner triangle outer diamond (ITOD) framework, therefore, represents an approach that is both comprehensive and relatable for medical educators. This model aims to guide the integration of technologies and innovations in a way that aligns with the broader goals of medical education.

## Introduction

The inner triangle outer diamond (ITOD) framework is presented in this work as a framework that considers the educational or learning environment as an ecosystem, especially when integrating innovative solutions and technologies in ways that could influence learning and practices within the educational ecosystem. Educational technologies or EdTech and innovations, including artificial intelligence, have supported learners and enhanced educators' delivery of quality medical education [1,2]. These initiatives have shown positive impacts, with reports highlighting their success in improving educational outcomes and shaping the future of medical education and practice. There is ample evidence that EdTech can enhance learner experiences, improve program outcomes, and prepare them for a tech-driven future. However, unintended consequences may arise when EdTech is not grounded in sound pedagogical principles or integrated effectively into the educational ecosystem. A holistic, systematic, and robust approach to introducing, deploying, and integrating EdTech and innovation for sustainable performance and excellence in medical and higher education requires considering the educational ecosystem. This approach enables educators to satisfy the fundamental requirements of adaptation, standardization, integration, and compliance when deploying EdTech and innovations [3,4].

The idea of an ecosystem approach is not new in medical education. Ecosystem-based frameworks address specific aspects of medical education, including Education for Sustainable Healthcare (ESH), which focuses on relationships among healthcare-related ecosystems as they relate to medical students' knowledge and attitudinal needs [5]; the Evidence Ecosystem concept, which highlights institutional policies and practices enabling feedback loops between practice and teaching [6,7]; and the Complex Systems and Ecosystem Thinking approach, which emphasizes reflective, contextual, integrative, and multidimensional education [8,9]. The One Digital Health framework underscores the integration of digital health and One Health at individual, population, and complex-ecosystem levels [10]. However, no major ecosystem-based framework has yet addressed EdTech and innovation deployment and integration, highlighting the significance of this article.

In this context, an educational ecosystem refers to the interconnected network of people, resources, technologies, policies, and practices that interact dynamically to support learning within and around an institution, such as a college, medical school, or university. The concept provides an opportunity to examine the basic components of the educational system from both structural and functional perspectives. While the "inner triangle, outer diamond" or ITOD framework of the educational ecosystem is not specifically premised on the ecological theory of systems (ETS) [11], it draws on the ecosystem concept as an effective means of functionally describing a system of interactive and interdependent components. The overall existence, functionality, and performance of the system depend not only on the performance of each component but also on the interactions between them and the quality of the governing dynamics.

The ITOD framework's inner core comprises a triad--curriculum, pedagogy, and assessment--forming a triangular sub-component of the ecosystem. The inner triangle provides a framework for the contexts within which primary educational activities occur, while the outer diamond components describe all other elements that functionally interact with the inner triangle. Together, they create a comprehensive and functional ecosystem capable of delivering effective and sustainable educational and training experiences. This work presents the ITOD framework with a learner-centered underpinning philosophy. The ITOD framework is presented to structurally and functionally represent the learning environment as an ecosystem with interacting and interdependent parts; and to further apply it for optimal integration of technologies and innovations. Its application could address the problems of poor technology integration, heterogeneities in EdTech applications, and negatively disruptive innovations [9,10].

## Technical report

The ITOD framework consists of three inner triangles and six outer diamond elements, as explained in the following sections.

The inner triangle

**Figure 1:**
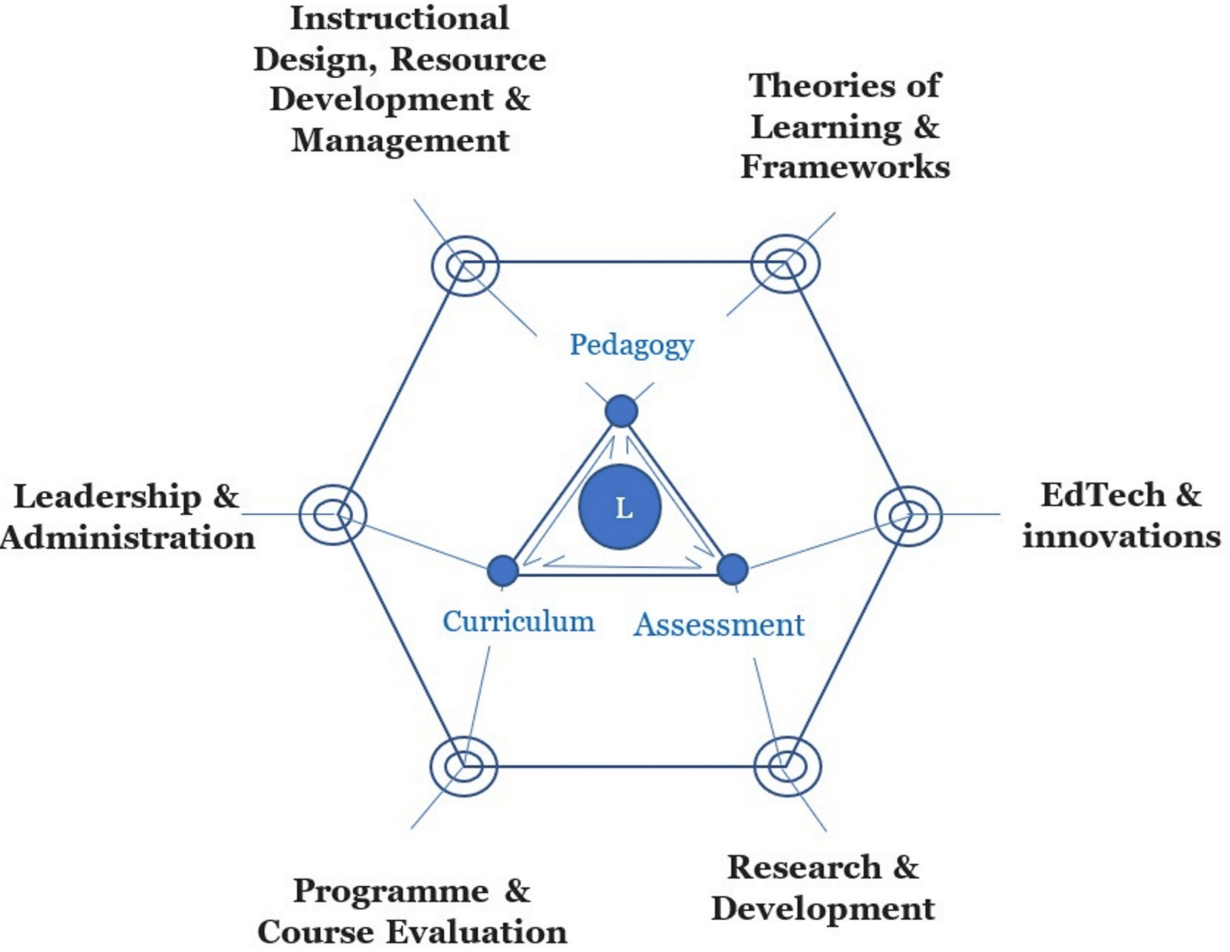
An illustration of the ITOD model shows the items that constitute the inner triangle and the outer diamond, including their contributions to the ecosystem. The connecting lines indicate their interactions. These interconnections and relationships, depicted by the connecting lines, illustrate the pattern of interaction between the ecosystem members to ensure synergy, internal equilibrium, and the overall performance of the system. Very importantly, the learner (L) is at the center of the system and its mission. ITOD: inner triangle and outer diamond; L: learner.

In the ITOD framework (Figure 1), the inner triangle consists of the curriculum, pedagogy, and assessment (Figure 1); these are aspects of medical education that primarily involve educators. The impacts of EdTech and innovations would also directly influence these components of the ecosystem. Notably, medical educators typically deploy Edtech and innovations for educational activities, nonetheless, the impacts of intervention and innovations are often measured and justified by the effect that they have on assessments as measures of learning and competences. Furthermore, the curriculum is what primarily determines what EdTech and innovations are deployed to accomplish. 

Inner Triangle Outer Diamond's (ITOD’s) Emphasis on the Curriculum

First, the curriculum serves as a template of instruction and, as such, could be described as a DNA of the educational program. The curriculum does not just present the content of what constitutes learning in the cognitive, psychomotor and affective domains, but also the indicative content mode of instruction outcomes that are expected of each or specific learning activities, as well as the overall competencies that a program should deliver. Regarding competencies, these are measures of functions, roles, or duties that graduates of the educational program are required to perform in their roles as workers and professionals. A quality curriculum is integral to the success and impact of a programme; hence, the use of EdTech and innovations should carefully and properly consider the programme curriculum [12,13]. ITOD’s emphasis on the curriculum requires that the use of EdTech or innovations properly satisfy curricular requires in its various dimensions.

*Inner Triangle Outer Diamond's (ITOD’s) Emphasis on Pedagogies* 

Pedagogies are specifically designed activities that facilitate learning and training, enabling the progressive acquisition of competencies in knowledge, skills, and attitudes. By the end of a program, these pedagogical activities ensure that graduates can perform their job roles as required. While instructional design focuses on determining how learning activities are structured and instructional content is delivered, pedagogies leverage learning theories, frameworks, and principles to guide educators or instructors in facilitating these activities effectively. This ensures that learners and trainees acquire education that meets established standards and fulfills their training goals, objectives, or outcomes for specific activities. Noting that learning can occur in various contexts, including classrooms, fields, laboratories, clinics, simulation (SIM) facilities, or workstations, it is crucial to recognize that effective learning can only be guaranteed when activities are designed in alignment with instructional design principles and delivered using sound pedagogical frameworks and practices. The proper application of pedagogical principles and frameworks would significantly contribute to success in efforts to deploy EdTech and innovations for medical education [14].

Inner Triangle Outer Diamond's (ITOD’s)​​​​​​​ Emphasis on Assessment

Assessment can be considered a means of testing fitness to perform and competence to achieve desired results. In medical education, the collection of assessments in the course of training could be illustrated as cumulative tests for ascertaining fitness for purpose. As such, it is impractical to guarantee that effective learning has occurred without adequate assessment. In medical and higher education, assessment serves as a standardized process for testing, validating, and affirming that learning outcomes have been achieved and that the competencies defining program deliverables have been acquired [15]. Therefore, the use of innovations and EdTech in an ecosystem should consider the assessment leg of the inner triangle or triad, ensuring that the use of such technologies and innovations satisfy proper, appropriate, and valid assessments that could truly and effectively measure and validate 'fitness for purpose'.

Connecting the Inner Triangle Elements

Considering the above, it is crucial that the triad of curriculum, pedagogy, and assessment is collectively and adequately addressed when designing instructional activities, facilitating learning, and determining whether competencies have been acquired. Furthermore, when innovations--whether in the form of resources, products, methods, or technologies--are introduced into the educational ecosystem, careful consideration must be given to these three core components of the educational ecosystem. It is worth noting that most innovations or innovative methods, as well as educational technologies directly used for medical and higher education activities, are often introduced through learning and training activities, thereby placing primary emphasis on pedagogical considerations. However, their potential impact and ability to meet program requirements can only be validated when they are appraised for their capacity to satisfy curricular requirements and deliver outcomes based on assessment standards. 

In instances where medical educators and clinical trainers introduce technologies and innovations through activities, such as those in classrooms, laboratories, or clinical environments, it becomes essential to triangulate and align these innovative products, methods, or educational technologies with their implications for curriculum requirements and assessment standards. The "inner triangle, outer diamond" model of the educational ecosystem emphasizes that educators and trainers seeking to introduce educational technologies and innovations into their programs must thoroughly consider the triad curriculum, pedagogies, and assessment requirements. For example, no matter how theoretically sound or pedagogically impressive a particular approach may appear, it might be challenging to justify its implementation if it fails to meet the requirements and tenets of the program curriculum or if it is inadequate in preparing learners and trainees for institutional board or council examinations or other competency-based assessments.

Outer diamond component of the inner triangle outer diamond​​​​​​​ (ITOD) framework

The outer diamond component of the ITOD framework consists of six parts as described in the following sections.

Leadership & Administration

The transformation of the medical and higher education ecosystem, even with technologies and innovation, and the direction of change rely significantly on quality leadership and administration [16,17]. In terms of the hierarchy of influence, among the members of the outer diamond part of the ecosystem, leadership occupies the top position as leaders are capable of initiating changes of various magnitudes, including those that can significantly alter the direction and trajectory of organizational progress and development. Administrators are largely responsible for maintaining equilibrium and outcomes within the scope of authority granted by leadership and the governing forces. These forces include those created by the internal dynamics resulting from the interdependent effects of the ecosystem's inherent members and forces. The other types of forces include those originating from the external universe, i.e., the larger environment within which the ecosystem is situated, which might include the government, society, and interested parties. The ITOD framework, therefore, emphasizes that leadership capacity to implement change, capacity to harness organizational resources, abilities to inspire collaboration and synergies, and, very importantly, purposeful interest in initiating or facilitating change would all be critical factors in leading significant changes through the use of technologies and innovations.

Program & Course Evaluation

Evaluation of programs provides insight into the performance of courses and programs, resulting from the interaction of members within the educational ecosystem [18,19]. Also, evaluation of programs is important for determining how well programs are implemented in line with their design and curriculum, as well as their capacity to meet expected outcomes, often marked by competencies and impacts. Proper course and programme evaluations are therefore integral to the improvement of courses and programs. As such, it would be a significant disservice to learners and other stakeholders if courses are not properly evaluated, and even more so if programs are not adequately evaluated to ensure they are performing optimally and with valid parameters. It must be emphasized that courses and programs should not be evaluated solely for external validation and approval, such as meeting the requirements of accreditors and regulators, but also to ensure that programs are meeting the expectations of all key stakeholders, including learners, educators, professional bodies, and the community as well. Valid methods and validated instruments should be used for evaluation. Furthermore, the collected data and information should be subjected to rigorous and thorough analytical procedures to yield objective, reliable, and applicable insights and inferences. Subjective interpretation of data with minimal statistical rigor might not significantly benefit the overall interests of the programs and stakeholders, especially learners. In this context, the ITOD framework does not just consider evaluation as an activity or process, but much more importantly, the entity, unit, and stakeholders that are responsible for evaluation in an educational ecosystem, collectively.

EdTech & Innovations

EdTech and innovations are key driving forces for change; in fact, innovations are catalytic in terms of their impact and the magnitude of the changes they cause. The most radical and wide-reaching changes are often driven by innovations, such as methods never used before, solutions not available in previous eras, or technologies that are new and products of evolution and advancements. Technologies are major driving forces of change and advancement in almost all walks of life in the post-modern world, and their influence is steadily growing. Therefore, educational technologies and innovations should be given their place and the required attention when considering the optimal performance of educational ecosystems, as well as in efforts to initiate change and advancement through technologies and innovations. It is noteworthy of mention that EdTech and innovations should be applied rigorously and with adherence to sound learning theories, pedagogical principles, and evidence to inform standard practice [20]. Again, the ITOD framework requires that the ecosystem unit concerned with EdTech and innovation integration plays optimally enabling roles in promoting and leading change with innovation.

Theories of Learning and Frameworks

Theories of learning, as well as frameworks, are very important in determining the selection of methods and pedagogical approaches. It is important for educators to note that every established method of instruction and pedagogical framework has guiding principles that are rooted in tested theories. Therefore, educators, while being dynamic and creative, need to understand the guiding principles and the foundational theories of instruction methods and pedagogical frameworks. This understanding enables them to adapt methods in creative ways to effectively enhance their learners' experiences. It is also important to mention that entirely new methods should be grounded in sound theories and supported by appropriate principles to avoid merely experimenting with learners' experiences. Learning theories and pedagogical frameworks are important for enshrining a culture of best practice in education [21,22]. As such, ecosystem units concerned with teaching or training methods and learning activities should contribute meaningfully to the ecosystem's culture of innovation.

Instructional Design, Resource Development & Management

Instructional design is a very important aspect of medical and higher education that is often relegated to the background or left to experts in the field. While educators do not need to be super experts in the field of instructional design, it is important that they understand and appreciate the guiding principles and relevant knowledge. Educators should also be informed enough to work with experts or established guidelines to design innovative instructional activities, such as classroom sessions and clinical teaching activities. Thankfully, most established pedagogical approaches have rich information in texts and scholarly literature about their guiding principles and successes with specific adaptations. Nevertheless, the subject of instructional design has become particularly important in the current era, where the use of innovations and technologies significantly influences the design of learning activities in almost every context [23-26]. The introduction of innovative and advanced technologies has the potential to change the dynamics of instruction within the educational ecosystem to such an extent that being well-grounded in relevant learning and instructional design theories and principles is essential. This foundation enables educators to make all necessary considerations and possible adjustments to other factors to ensure a healthy balance and guide the entire system toward optimal performance and sustainable quality practices. Therefore, the ITOD framework requires adequate contributions from the educational ecosystem unit concerned with instructional design and creation or curation of educational resources to make adequate contributions to the process of integrating and optimizing EdTech and innovations.

Research and Development

Research and development (R&D) has always been central to scholarship and advancements in best practices. Nevertheless, medical and higher education are not exempt from the increasing demand for empirical evidence to inform best practices, especially in light of the rapid advances and changes brought about by innovations and the introduction of various technologies [21,27]. To this end, educators need to specifically design research activities to test their new or innovative methods and validate practical teaching techniques and innovations. This allows them to obtain objective and well-analyzed data to inform best practices. Furthermore, evidence needs to be systematically determined from diverse backgrounds to guide what should constitute best practices within communities of practice and academic integration. More than ever before, educators need to harness evidence from experimental uses of innovations and technologies to assess their impact, improve experiences, and validate what could serve as references for best practices. Based on the ITOD framework, the ecosystem unit concerned with research and resource development is required to make significant contributions to ensure a healthy approach to driving sustainable innovation in the educational ecosystem.

## Discussion

This section addresses the applications of the ITOD framework with a special emphasis on the integration of its parts and the use of specific cases to illustrate its practical applications.

Integration

**Figure 2:**
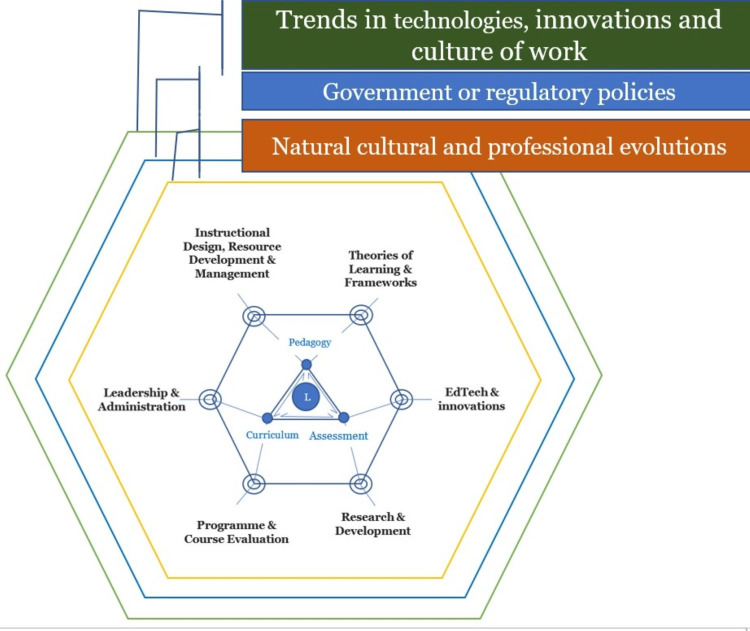
The ITOD framework illustrating the inner triangle and outer diamond components, and external centripetal forces influencing the ecosystem. The ITOD framework illustrating the inner triangle and outer diamond components, and external centripetal forces influencing the ecosystem, which include (1) trends in technologies, innovations and culture of work; (2) government or regulatory policies; and (3) natural cultural and professional evolutions. L: learner; ITOD: inner triangle outer diamond.

It is important not only to consider the individual components of the ITOD framework (Figure 1), but also to evaluate their integration and the dynamics that characterize the interactions between the members of the ecosystem. The ideal foundational perspective from which to consider this framework is a learner-centered approach, whereby the entire ecosystem is driven toward making the trainee competent through a competency-driven, learner-centered approach to educational activities and processes. While it is important to define each component of the ecosystem, highlight their roles, and properly define their functions to ensure they effectively contribute to the ecosystem, it is also essential to ensure that the members of the entire ITOD ecosystem relate and interact effectively for optimal performance of the overall system. The functional synergy of the ecosystem relies on both the functionality and integration of each member of the inner triangle and the outer diamond, as well as the interactions between them. Notably, while the ecosystem construct primarily focuses on the educational ecosystem, which could be a school, college, or university, etc., it also recognizes the fact that external centripetal forces are also exerting forces of influence on the ecosystem. Therefore, the stakeholders that play specific roles within the inner triangle and outer diamond should put such external forces into proper perspective and give them due consideration (Figure 2).

Case studies and practical applications of the inner triangle outer diamond (ITOD) framework

It is crucial to adopt a systems-thinking, ecosystem-based philosophy when implementing strategic changes in medical education--which the ITOD framework promotes and supports. Examples of innovative and strategic educational projects are those involving artificial intelligence, virtual and augmented realities, simulation-based medical education (SBME), and related technologies. Selected relevant reports on the use of major innovations and EdTech that could serve as case studies and practical examples to illustrate ITOD applications are considered in the next sections. In these instances, efforts to connect the various elements of the ITOD framework are illustrated, with emphasis on key highlights and factors that could affect sustainability.

*Case 1-Inner Triangle Outer Diamond* *(ITOD) Framework Application to the Use of Virtual and Augmented Realities (VR and AR)*

In a case where virtual reality was applied to undergraduate medical education--with reported success and evidence supporting many aspects of the ITOD model--the efforts to integrate technology and innovation can be clearly aligned with the framework. Specifically, a virtual reality platform called Immersive Virtual Anatomy Laboratory (IVAL) was deployed to teach anatomy, with a focus on the musculoskeletal system [28]. There is evidence that the project applied relevant pedagogical and technological principles to the use of IVAL, thereby satisfying the ITOD model’s educational requirements. Furthermore, IVAL’s deployment aligned with learners’ needs in the curriculum component of ITOD’s inner triangle. In their initial report, the authors emphasized data collection spanning both research and evaluation--key elements of the outer diamond domain. They also addressed core learning aspects dictated by curricular and programme requirements by conducting a comparative impact analysis of IVAL versus traditional instruction. Outcomes assessed included learning time, completion time, and accuracy scores. It was evident that the project thoughtfully considered all requirements of ITOD’s inner triangle (Figure 1). Such rigorous alignment and evaluation will undoubtedly support the method’s viability and scalability within existing programmes and in other educational contexts.

In an effort to deploy the IVAL innovation more effectively, the authors considered knowledge retention and technology acceptance, emphasizing that this occurred within a collaborative virtual-reality-based medical education context [29]. This implies that the authors possessed--if only implicitly--the leadership capacity to guide this type of innovative change. Specifically, the approach reportedly adhered to learning theories rooted in constructivism, situated learning, and problem-based learning [28-30]. It also addressed key elements of the outer domain of the ITOD framework, including the following: 1. Theories of learning and framework development; 2. Appropriate selection and deployment of EdTech and related innovations; 3. Emphasis on research and development; 4. Programmatic and course evaluation; 5. Effective leadership and administration; and 6. Instructional design, resource development and management. One area needing further attention is the explicit establishment of processes for using and managing the IVAL innovation to ensure long-term sustainability. Although there is initial evidence of quality leadership during the experimental phase of this change, capacity-building for sustainability is required at all leadership levels and throughout actual practice.

As presented in this case, virtual reality and simulation technologies represent some of the most significant advances currently reshaping medical education, indicative of the presence of external centripetal influences (Figure 2). These innovations demonstrate the intricate challenges involved in incorporating educational technology, presenting substantial opportunities to improve learning throughout the medical training environment. Through immersive and interactive experiences, VR-based simulations have the capacity to transform teaching methodologies, moving beyond conventional educational approaches [28]. These technologies influence curriculum development by facilitating the design of innovative training programs for intricate clinical skills and anatomical studies [29]. Additionally, implementing VR and simulation requires thoughtful development of evaluation methods to accurately gauge student achievement within these engaging digital environments [30]. Nevertheless, effective implementation demands a comprehensive systemic perspective that extends beyond the technology components to include instructor training, budget considerations, ethical implications, and coordination with institutional objectives. Consequently, the ITOD framework offers medical educators a strategic tool for planning, executing, and assessing the incorporation of VR, simulation, and other sophisticated educational technologies into their academic programs in line with the ecosystem philosophy.

Case 2-Inner Triangle Outer Diamond (ITOD) Framework Application to Simulation-Based Medical Education (SBME)

The Boston Children’s Hospital immersive simulation concept and approach is a prime example of a systematic method for integrating innovation into medical education, with a special focus on problem-based training and clinical practice. In terms of alignment with the ITOD innovation-triangle training framework, the center has developed a well-defined programme and template for what it terms “immersive simulation” activities, thereby satisfying a key curricular requirement. Rather than shoehorning this template into a single medical programme or discipline, the facility and its offerings form a flexible, adaptable ecosystem capable of delivering tailored training and instruction. Authors affiliated with the center also developed and published the SIM-Zone concept, which underpins tiered levels of simulation based on learner need [31]. This innovative approach provides a structured, forward-looking curricular model for simulation-based medical education. Moreover, by leveraging both technology and pedagogical innovation, the model allows for the concurrent development of adapted curricula that align with broader programme objectives. The instructional design further incorporates mechanisms for pre-assessment of learner competence, linked explicitly to SIM-Zone levels and defined learning goals.

In terms of the outer diamond considerations, the extent to which the SIM-zone concept aligns with relevant learning theories is directly responsible for the feasibility, impact, and sustainability of its programs. There has been a significant deployment of innovations and technologies, which explains why the center can offer diverse simulation activities. Publications from the center have detailed its programs, methods, approaches, and impacts--of which the SIM-Zone model and its underpinning philosophy represent a major contribution to advances in simulation-based medical education [32,33]. The center has established procedures and protocols for measuring outcomes and validating practices. Strong leadership and clear administrative support have driven its numerous partnerships and collaborations. Moreover, the development of a unique and innovative curricular pathway in the SIM-Zone--and other related concepts--adheres to the ITOD tenet of intentional design. Similarly, the impact and sustainability of the Boston Children’s Hospital’s immersive simulation program can be linked to its emphasis on an ecosystem approach to leading change with innovation, as illustrated in this report. This model of technology integration and change leadership may serve as a suitable template in clinical and professional education contexts, where interventions must be well-designed, impactful, sustainable, and transformative.

Case 3-Inner Triangle Outer Diamond (ITOD) ​​​​​​​Framework Application to the Use of a Standalone EdTech, the Anatomage Table

The use of the Anatomage table to complement cadaveric dissection, with reported evidence of success, could serve as a good example of technology integration at the level of pedagogy, a component of the inner triangle [34,35]. As reported, this use of the Anatomage table for virtual dissection was indicated in the curriculum with appropriate consideration for learning objectives to be covered, including time allocation, as well as the method of facilitation. For this, a protocol was developed to guide sessions. This would satisfy the requirement of adequate curricular consideration. In this reported case, despite the fact that the standalone innovation or EdTech was introduced for pedagogical purposes, the curriculum was properly considered. In terms of the assessment, low-stakes assessment of learning was conducted with the same technology, using its assessment features following learning activities. These were formative or low-stakes assessments. For summative assessment or end-of-course exams, questions from the technology-enabled sessions were presented to learners as a proportionally representative component of the total examination. Thus, learning with the use of technology was tested through both summative and formative assessments. Thus, the reported example indicated proper consideration for curriculum, pedagogy, and assessment being key to the inner triangle condition of the ITOD framework. This approach ensured the standardization and sustainability of practices at all levels: executive leadership (as represented by the dean's office); departmental leadership (led by the head of department); and the instructional/didactic level (managed by the director of the simulation facility). There was also consideration given to consistency in practices [34-36].

In addition to the inner triangle considerations, the outer diamond conditions were also given due attention. The aspect of learning theories and frameworks was addressed by choosing a virtual dissection approach aligned with adult learning theory tenets. To model the traditional pedagogy of anatomical dissection--as originally indicated in the curriculum--a technology offering true-to-life, virtual representations of human anatomy (rather than artificial constructs) was selected: namely, the Anatomy table. A thorough review of the literature was first conducted to identify best practices for adapting this technology to a medical-school curriculum, evaluating its merits, limitations, and critical considerations. Also, during active sessions, data were then collected not only on users’ engagement but also on learners’ competencies and attitudes toward the technology. After an initial successful deployment, further research was undertaken within the institution's internal ecosystem and subsequently benchmarked against national and international implementations [37,38]. Through systematic course and programme evaluation, iterative adjustments were made until a standardized protocol was established for the technology implementation, development, and integration into the curriculum.

There was quality academic leadership with competence and capacity to lead the process of change with EdTech integration and with coordinated effort from the team. Throughout this process, leadership demonstrated the capacity to lead change and continually promote innovations. Leadership at various levels collaborated to design and advance an innovation-and-change agenda, supported by the necessary infrastructure and systems. Leaders contributed content expertise, pedagogical guidance, and technological competence to integrate the technology effectively and optimize its use. At the outset, resources were committed to orienting and training primary users via workshops, self-directed study, and hands-on sessions. The implementation team then collaborated to design an instructional model that embedded the new EdTech into specific courses and subject contexts, applying it both to protocol development and to the facilitation of actual learning sessions.

Case 4-Inner Triangle Outer Diamond​​​​​​​ (ITOD) ​​​​​​​Framework Application to the Use of AI in Medical Education

It is intriguing to note how challenging it remains at the moment to find an AI-integration approach with a coherent ecosystem approach for its agenda and deployment. Evidently, educators are dedicating earnest efforts to leverage AI in multiple domains-pedagogy (e.g., to support teaching and learning), assessment, and curriculum review, development, or enhancement--while striving to align curricular content objectively with actual instruction [39,40]. Nevertheless, proper integrations that preserve the core intent of AI deployment are not yet commonplace. Although many educators are experimenting with AI to optimize learning activities and educational processes, limitations in data availability and evidence on best practices persist. The ITOD framework could serve as the foundation for a robust model to guide effective and responsible AI implementation and integration.

Consistent with its tenets, it would be beneficial for educators seeking to deploy AI to consider the following: 1. Clearly define their intended points of use and anticipate how AI applications might impact other core ITOD activities. 2. Ground their implementations in established learning theories and instructional frameworks, particularly when AI is used for direct teaching or assessment. 3. Apply rigorous research and evaluation methods to validate AI-enabled innovations and inform iterative refinement. 4. Ensure programmatic and course-level validation to support scalability and sustainability. 5. Cultivate quality leadership with expertise in change management and technological competencies. 6. Adhere to sound instructional-design principles and resource-management practices to maximize the pedagogical benefits of AI. By systematically addressing these considerations, medical education programs can harness AI’s potential while maintaining alignment with the ITOD framework’s standards for innovation, pedagogy, and sustainability.

## Conclusions

The ITOD framework can support educators and stakeholders in the medical and higher education systems to work with a structurally representative and functionally operational framework. It can guide their educational initiatives and the use of innovations, including technologies, for educational activities. This framework is presented in its original version and is not presumed to be universally infallible when applied to various contexts. A current limitation is that the ITOD framework has not yet been tested through extensive empirical research using a subjective inductive approach. Users are encouraged to continuously report experiences following its deployment or the outcomes of experimental and pilot applications in efforts to further refine the framework for optimal future use. Expectedly, this framework would be subjected to continuous scholarship efforts and critique. It is noteworthy that the ITOD framework could guide educators who are primarily scientists, content experts, and clinicians, but with limited competencies in educational theories, pedagogical frameworks, and instructional design, to employ a well-constructed framework in guiding their efforts to adopt and apply innovations and technologies. In addition, it would help educators to objectively evaluate or ensure optimal impacts of their innovations or technologies.
